# The complete chloroplast genome sequence of *Artocarpus gomezianus* (Moraceae) from Xishuangbanna, China

**DOI:** 10.1080/23802359.2021.1973603

**Published:** 2021-09-09

**Authors:** Xinggu Lin, Tao Lin, Sun Zhixia, Arief Priyadi

**Affiliations:** a Key Laboratory of Plant Resources Conservation and Sustainable Utilization, South China Botanical Garden, Chinese Academy of Sciences, Guangzhou, China; b Fujian Provincial Key Laboratory of the Development and Utilization of Bamboo Resources, Sanming University, Sanming, China; c Bali Botanic Garden - Research Center for Plant Conservation and Botanic Gardens, Indonesian Institute of Sciences (LIPI), Bali, Indonesia

**Keywords:** *Artocarpus gomezianus*, chloroplast genome, medicinal plants

## Abstract

*Artocarpus gomezianus* is a medicinal species native to Asia. To infer its phylogenetic relationship to the other Moraceae, the complete chloroplast genome of *A. gomezianus* was sequenced. The whole chloroplast genome is 160,743 bp in length, consisting of a pair of inverted repeat (IR) regions of 25,691 bp, one large single-copy (LSC) region of 89,241 bp, and one small singlecopy (SSC) region of 20,120 bp. The overall GC content of the complete chloroplast genome is 35.81%. Maximum likelihood analysis using 11 complete plastomes of the Moraceae and *Cannabis sativa* (Cannabaceae) designated as the outgroup, resolved *A. gomezianus* in a clade with *A. petelotii* and *A. hypargyreus*. These phylogenetic results are not consistent with previous findings based on nuclear loci in which *A. gomezianus* was grouped as a sister to a clade containing *A. petelotii* and *A. hypargyreus*. The complete chloroplast genome of *A. gomezianus* will provide a powerful tool to accelerate pharmacological development, systematics, and future phylogenetic studies in the Moraceae.

*Artocarpus gomezianus* Wall. ex Trécul is a plant species with a variety of medicinal values, among approximately 70 other species classified to the genus *Artocarpus* (Moraceae) (Williams et al. 2017). The arylbenzofurans, flavonoids, phenolics and stilbenoids extracted from *A. gomezianus* are α-glucosidase inhibitors (Nuntawong et al. 2019) and tyrosinase inhibitors (Likhitwitayawuid et al. 2000; Likhitwitayawuid and Sritularak 2001), show antiherpetic activities (Likhitwitayawuid et al. 2005, 2006). A facile and eco-friendly combustion method mediated by *A. gomezianus* fruit was applied to synthesize spherical nanoparticles of zinc oxide, which were used as anticancer, antibacterial and antifungal materials (Anitha et al. 2018). No genomic resources have been reported for this species, and this hinders the development of *A. gomezianus* pharmacological properties. In the present study, we report the complete chloroplast genome sequence of *A. gomezianus* to contribute to further phylogenetic and population genetic studies.

The voucher specimen of *A. gomezianus* was collected from Menglun Town, Mengla County of Xishuangbanna (Yunnan, China; Long. 101.2742 E, Lat. 21.9198 N; Altitude: 555.35 m), and is deposited at the herbarium of South China Botanical Garden, Chinese Academy of Sciences (contact person: Shuangwen Deng, email: dengshuangwen@scbg.ac.cn, accession number: SCBG-BN-26). The DNA was extracted from fresh young leaves of *A. gomezianus* following the modified CTAB-chloroform protocol (Doyle and Doyle 1990). The DNA was sequenced on an Illumina Hiseq 2000 platform at Novogene-Beijing (Illumina, San Diego, CA) and generated 4.5 Gb raw data. The chloroplast genome sequence was assembled using the default settings in NOVOPlasty (Dierckxsens et al. 2017). A ribulose-1, 5-bisphosphate carboxylase/oxygenase (*rbcL*) gene sequence from *A. heterophyllus* (GenBank accession no. MK303549) served as the seed sequence, and the complete chloroplast genome sequence of *A. heterophyllus* was used as a reference to resolve the inverted repeat in the chloroplast genome of *A. gomezianus*. The assembled chloroplast genome was annotated using PGA (Qu et al. 2019) and GeSeq (Tillich et al. 2017), and also adjusted manually. The annotated chloroplast genomic sequence was deposited in GenBank under the accession number: MW837773.

The chloroplast genome of *A. gomezianus* is 160,743 bp in size, displays a 35.81% GC content, and includes a characteristic quadripartite structure with a LSC of 89,241 bp, an SSC of 20,120 bp and a pair of IRs of 25,691 bp. A total of 131 genes were identified, including 85 protein-coding, 38 tRNA and 8 rRNA genes. The chloroplast genome length of *A. gomezianus* is 266 bp, 209 bp smaller than that of *A. petelotii* (161,009 bp, MW250918) (Chen and Liu 2021) and *A. hypargyreus* (160,952 bp, MN720648) (Li et al. 2020), but 356 bp larger than that of *A. heterophyllus* (160,387 bp, MG434693) (Liu et al. 2017). The GC content and genome organization of *A. gomezianus* is similar to that of *A. petelotii* and *A. hypargyreus*.

To confirm the phylogenetic position of *A. gomezianus*, the complete plastome sequences of ten previously published Moraceae species, including *A. camansi*, *A. champeden*, *A. heterophyllus*, *A. hypargyreus*, *A. petelotii*, *Morus indica*, *M. notabilis*, *Ficus carica*, *F. religiosa* and *Broussonetia papyrifera*, and one outgroup *Cannabis sativa* (Cannabaceae) were downloaded from the NCBI GenBank database. Complete sequences were aligned using the default settings in MUSCLE (Edgar 2004), and the phylogenetic tree (Figure 1) was constructed using the TVM + F+R2 model in IQ-TREE (Nguyen et al. 2015). Based on 1000 bootstrap replicates, the phylogenetic tree strongly supported that *A. gomezianus* is placed in a clade with *A. petelotii* and *A. hypargyreus* (Figure 1). However, in the phylogenetic framework constructed from 517 genes, *A. gomezianus* was sister to the clade containing *A. petelotii* and *A. hypargyreus* (Gardner et al. 2021). It suggests that the chloroplast of *A. gomezianus* may share close ancestry with that of *A. petelotii*, although they are clearly distinct in nuclear DNA sequences. In conclusion, the *A. gomezianus* chloroplast genome reported here will provide a solid foundation for phylogenetic and evolutionary studies in *Artocarpus* and may improve the detection of various molecular mechanisms of the medicinal properties found in *A. gomezianus*.

**Figure 1.**
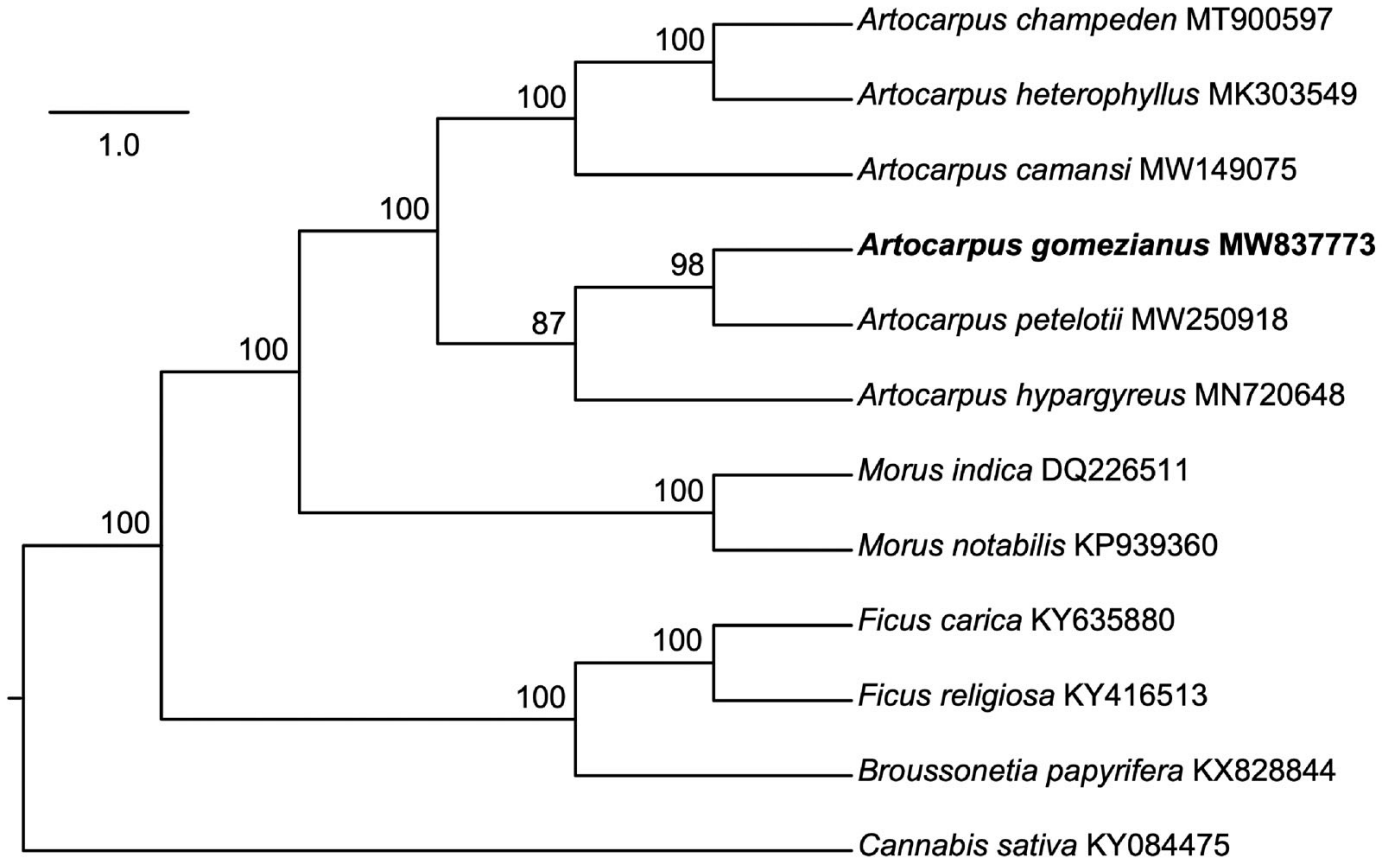
Maximum-likelihood tree showing the relationship among *A. gomezianus* and ten other species classified in the Moraceae and one outgroup taxon (*Cannabis sativa*), using complete chloroplast gene sequences. Bootstrap supports based on 1000 replicates are given at the nodes.

## Data Availability

The data is available in GenBank of NCBI at https://www.ncbi.nlm.nih.gov/, under the accession number [MW837773]. The associated BioProject, SRA, and Bio-Sample numbers are PRJNA717896, SRS8590981, and SAMN18515340 respectively.
